# Clinicopathological Perspectives of Liver Mass Biopsies: A Single Center Experience of 406 Cases

**DOI:** 10.34172/aim.34218

**Published:** 2025-06-01

**Authors:** Ömer Atmış, Hanife Seda Mavili, Fatma Seher Pehlivan, Ahmet Burak Ağaoğlu, Atike Pınar Erdoğan, Mustafa Faraşat, Semin Ayhan

**Affiliations:** ^1^Department of Pathology, Faculty of Medicine, Manisa Celal Bayar University, Manisa, Turkey; ^2^Department of Medical Oncology, Faculty of Medicine, Manisa Celal Bayar University, Manisa, Turkey; ^3^Department of Radiology, Faculty of Medicine, Manisa Celal Bayar University, Manisa, Turkey

**Keywords:** Cholangiocarcinoma, Hepatocellular carcinoma, Immunohistochemistry, Liver mass biopsy, Liver metastasis

## Abstract

**Background:**

The increasing use of imaging techniques has led to a rise in the detection of liver masses, making it crucial to accurately diagnose their nature. While advances in radiology have reduced the need for liver biopsy in hepatocellular carcinoma (HCC), biopsy remains essential fo r diagnosing various liver lesions, including metastatic tumors. This study aims to evaluate the diagnostic role of liver core needle biopsies, with a particular focus on identifying the primary tumor in cases of liver metastases with an unknown primary.

**Methods:**

We reviewed a total of 406 liver core needle biopsies performed for liver masses between 2017 and 2022. Clinical, radiological, histopathological and immunohistochemical (IHC) data for primary and metastatic tumors were evaluated.

**Results:**

Of the 406 liver biopsy cases, a significant portion were diagnosed as metastatic lesions, with common primary sites identified as gastrointestinal (GI), lung, and breast cancers. IHC markers showed varying positivity rates across different tumor types, with GATA-3, CDX2, and TTF1 proving particularly useful in distinguishing the tumor origin. While some markers were highly specific, others exhibited variable expression, highlighting the complexity of diagnosing metastatic tumors with unknown primaries.

**Conclusion:**

Liver biopsy remains a crucial diagnostic tool in identification of primary and metastatic liver tumors, especially when the primary site is unknown. IHC analysis enhances the accuracy of diagnosis, though it should be used in conjunction with clinical and radiological data. This study underscores the importance of a multidisciplinary approach in managing liver masses, with further research needed to optimize diagnostic strategies and improve patient outcomes.

## Introduction

 With the increased use of imaging methods in the diagnosis of abdominal symptoms, the detection frequency of liver masses has also risen.^1^ Accurate and reliable determination of the nature of liver masses is critical not only to reassure individuals with benign lesions but also, and perhaps more importantly, to ensure the correct diagnosis of malignant lesions and to determine appropriate treatment approaches for patients.^1^ Although the use of liver biopsy in the diagnosis of hepatocellular carcinoma (HCC) has decreased due to advances in radiological techniques, liver biopsy remains a significant diagnostic option in the management of various other liver lesions.^2^ The differential diagnosis of liver masses spans a broad spectrum, ranging from liver abscesses to benign tumors and cystic lesions, primary malignant tumors, and metastatic malignancies.^2^ The liver is a common site for metastatic tumors; however, there is limited information on the frequency of diagnosis of tumors with liver metastases.^3^ Organotropism in liver metastases is influenced by factors such as blood flow, tumor stage, and histological subtype.^4^ Gastrointestinal (GI) cancers tend to metastasize to the liver due to the portal venous circulation.^4^ Liver biopsies performed for masses are essential not only for diagnosing primary liver tumors but also for differentiating between metastasis and a second primary tumor in patients with a known primary tumor. Moreover, in cases of liver metastases with an unknown primary tumor, liver biopsy facilitates the identification of the tumor’s primary origin.

 This study aims to report the diagnoses of core needle biopsies performed on detected liver masses, to determine the contribution of immunohistochemical (IHC) data in differentiating between primary and metastatic lesions, to investigate the role of liver biopsy in identifying the primary tumor in cases of metastatic liver tumors with an unknown primary, and to highlight the challenges encountered in determining the tumor’s origin.

## Materials and Methods

**Figure 1:**
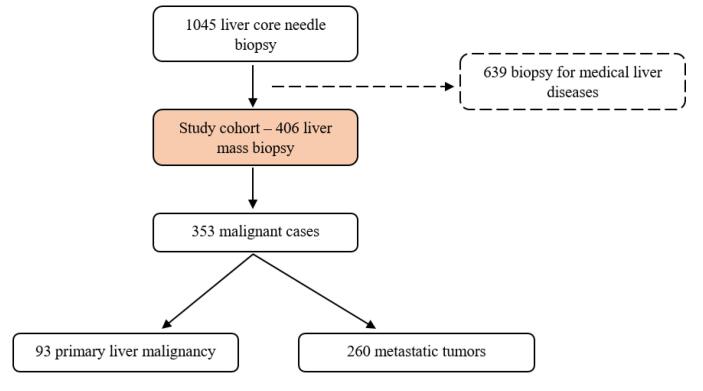
Diagram of Cases İncluded in the Study

 In this study, we evaluated liver core needle biopsies performed between 2017 and 2022. Of the 1045 liver core needle biopsies performed during this period, we included 406 cases with biopsies performed due to liver masses, while cases related to medical liver diseases were excluded (Figure 1). Radiological and clinical data of all cases were obtained from the hospital system. In metastatic cases, liver biopsies were considered to have a known primary if the primary tumor was identified radiologically or histopathologically at the time of the liver biopsy. Tumor size was determined by reviewing radiological data, and for patients with multiple masses, the diameter of the largest tumor was recorded as the tumor size.

 Statistical analysis was performed using IBM® SPSS® (version 25.0). Descriptive statistics were used to summarize patient demographics and tumor characteristics; continuous variables were reported as means, medians, and ranges, while categorical variables were presented as frequencies and percentages. The association between categorical variables, such as the distribution of single versus multiple lesions in primary versus metastatic tumors, was evaluated using the chi-square test. The Mann-Whitney U test was applied to compare tumor sizes between primary and metastatic lesions, as the data did not conform to a normal distribution. For survival analysis, the start time was defined as the date of the liver core needle biopsy, and the end time was defined as either the date of death or the date of the last follow-up, whichever occurred first. Follow-up data were collected from hospital records and national health database records. Survival analysis was conducted using the Kaplan-Meier method, and differences in survival distributions between groups were assessed using the log-rank test. Median overall survival times and their 95% confidence intervals were calculated, and the standard errors were reported.

 A Cox proportional hazards regression analysis was performed to evaluate the effect of multiple variables on overall survival. In the multivariable Cox models, the following covariates were included: age, sex, number of liver lesions (single/multiple), tumor size, and tumor origin (primary vs metastatic). Additionally, separate Cox regression analyses were conducted for specific subgroups including primary liver tumors (HCC and cholangiocarcinoma), metastatic tumors, HCC only, and CCA only. Variables that were statistically significant in univariate analysis or considered clinically relevant were included in multivariable models. Hazard ratios (HRs), 95% confidence intervals (CIs), and p-values were reported for each covariate.

 Results were regarded as statistically significant when *P* < 0.05. The study was approved by the institutional ethics committee.

## Results

 Of the 406 cases included in the study, 243 (59.9%) were male, and 163 (40.1%) were female. The age of the patients ranged from 1 to 88 years, with a mean age of 62.56 years and a median age of 64 years. Among the four patients under 18 years of age, the diagnoses were hepatoblastoma, fibrolamellar HCC (two cases), and metastatic solid pseudopapillary neoplasm of the pancreas.

 Of the total cases, 12 (3%) showed normal liver tissue, and 24 (5.9%) had inflammation or necrosis (excluding tumor necrosis). There were seven (1.7%) cases of benign liver tumors or mass-forming lesions (hemangioma, hepatocellular adenoma, and focal nodular hyperplasia), and one case (0.2%) of high-grade dysplasia. Cirrhosis without neoplastic lesions was identified in seven (1.7%) cases, and one case (0.2%) was diagnosed with IgG4-related disease. Among the 45 cases classified as non-neoplastic, eight (2%) were benign neoplastic lesions. One case (0.2%) showed tumor necrosis without viable tumor cells observed in serial sections. The remaining 353 cases (86.9%) were classified as malignant. The distribution of tumor types in malignant cases is shown in Table 1. Adenocarcinoma was the most common tumor type, observed in 222 (62.9%) of the malignant cases. Of the 353 malignant tumors, 93 (26.3%) were primary liver tumors, and 260 (73.7%) were metastatic tumors (Figure 1). The origins of the metastatic tumors are shown in Table 2. The most frequent sources of metastases were the lung and pancreas (49 cases each). The primary malignancies of the liver included 48 (51.6%) cases of cholangiocarcinoma, 44 (47.3%) cases of HCC, and one (1.1%) case of hepatoblastoma.

**Table 1:** Distribution of Tumor Types in 353 Cases Diagnosed with Malignancy

**Tumor Type**	**Number of Cases (%) **
Adenocarcinoma (including 48 CC cases)	222 (62.9%)
Hepatocellular carcinoma	44 (12.5%)
Neuroendocrine carcinoma	32 (9.1%)
Squamous cell carcinoma	11 (3.1%)
Lymphoma	7 (2.0%)
Neuroendocrine tumor	6 (1.7%)
Serous carcinoma	6 (1.7%)
Malignant melanoma	5 (1.4%)
Solid pseudopapillary neoplasm	4 (1.1%)
Urothelial carcinoma	4 (1.1%)
Renal cell carcinoma	3 (0.8%)
Sarcoma/GIST	3 (0.8%)
Hepatoblastoma	1 (0.3%)
Mesothelioma	1 (0.3%)
PNET	1 (0.3%)
Undifferentiated carcinoma	3 (0.8%)
Overall	353 (100%)

CC: Cholangiocarcinoma, GIST: Gastrointestinal stromal tumor, PNET: Primitive neuroectodermal tumor.

**Table 2:** Distribution of Metastatic Tumors According to Organs and Systems and Mortality Status of These Cases

**Organ**	**Number of cases (%)**	**Ex**	**Alive**	**System**	**Number of cases (%)**	**Ex**	**Alive**
Colon	37 (14.2%)	31 (83.8%)	6 (16.2%)	Gastrointestinal tract	48 (18.5%)	41 (85.4%)	7 (14.6%)
Stomach	8 (3.1%)	7 (87.5%)	1 (12.5%)				
Small intestine	2 (0.8%)	2 (100.0%)	0 (0.0%)				
Esophagus	1 (0.4%)	1 (100.0%)	0 (0.0%)				
Pancreas	49 (18.8%)	44 (89.8%)	5 (10.2%)	Pancreatobiliary system	53 (20.4%)	48 (90.6%)	5 (9.4%)
Gallbladder	4 (1.5%)	4 (100.0%)	0 (0.0%)				
Lung	49 (18.8%)	47 (95.9%)	2 (4.1%)	Respiratory system	50 (19.2%)	48 (96.0%)	2 (4.0%)
Larynx	1 (0.4%)	1 (100.0%)	0 (0.0%)				
Breast	40 (15.4%)	26 (65.0%)	14 (35.0%)	Breast	40 (15.4%)	26 (65.0%)	14 (35.0%)
Tuba/ovary	7 (2.7%)	5 (71.4%)	2 (28.6%)	Female genital system	10 (3.8%)	8 (80.0%)	2 (20.0%)
Endometrium	2 (0.8%)	2 (100.0%)	0 (0.0%)				
Cervix	1 (0.4%)	1 (100.0%)	0 (0.0%)				
Bladder	4 (1.5%)	4 (100.0%)	0 (0.0%)	Urinary system	9 (3.5%)	9 (100.0%)	0 (0.0%)
Kidney	4 (1.5%)	4 (100.0%)	0 (0.0%)				
Prostate	1 (0.4%)	1 (100.0%)	0 (0.0%)				
Skin	4 (1.5%)	2 (50.0%)	2 (50.0%)	Skin	4 (1.5%)	2 (50.0%)	2 (50.0%)
Peritoneum	1 (0.4%)	1 (100.0%)	0 (0.0%)	Mesothelium	1 (0.4%)	1 (100.0%)	0 (0.0%)
Lymphoma	7 (2.7%)	7 (100.0%)	0 (0.0%)	Lymphoid system	7 (2.7%)	7 (100.0%)	0 (0.0%)
Unknown primary	38 (14.6%)	38 (100.0%)	0 (0.0%)	Unknown primary	38 (14.6%)	38 (100.0%)	0 (0.0%)
Overall	260 (100%)	228 (87.7%)	32 (12.3%)	Overall	260 (100%)	228 (87.7%)	32 (12.3%)

###  Radiological Data

 Of the 353 patients with malignant tumors, radiological data on the number of lesions were available for 341 cases (92 primary tumors, 249 metastatic tumors). Single lesions were identified in 43 (46.7%) primary tumors and 30 (12%) metastatic tumors, while multiple lesions were observed in 49 (53.3%) primary tumors and 219 (88%) metastatic tumors. Statistical analysis revealed that metastatic tumors were significantly more likely to present as multiple lesions than primary tumors (Chi-square; *P* < 0.001). Radiological data on tumor size were available for 317 of the 353 malignant tumors. The mean tumor size was 7.83 cm, and the median was 8 cm (range: 1.5-19 cm) for the 89 primary tumors. For the 228 metastatic tumors, the mean size was 5.56 cm, and the median was 4.2 cm (range: 1-20 cm). Primary tumors were found to be significantly larger than metastatic tumors (Mann-Whitney U test; *P* < 0.001).

 Excluding seven cases of lymphoma (none of which were primary liver lymphoma), 189 (74.7%) of the 253 metastatic tumors had a known primary site at the time of liver biopsy, while 64 (25.3%) did not. Following liver biopsy, primary tumor was identified in 23 (36%) of these 64 cases. The identified primary sites were lung (9 cases), colon (7 cases), stomach (3 cases), pancreas (2 cases), ovary (1 case), and gallbladder (1 case). The primary tumor was found after liver biopsy in 10 (20.8%) of the 48 GI tract-derived tumors and in 9 (18%) of the 50 respiratory system-derived tumors.

###  Survival Analysis

 Among the 353 malignancy cases, 299 (84.7%) had died, and only 54 (15.3%) were alive (Table 2). The median follow-up time was 8 months, with an interquartile range (IQR) of 2 to 27 months. The average survival was calculated as 28.7 months. Notably, all cases with a follow-up duration shorter than 12 months had died within the first 12 months.

 Among the 93 patients with primary liver tumors, 71 (76.3%) had died (follow-up period: 1-80 months), while among the 260 patients with metastatic tumors, 228 (87.7%) had died (follow-up period: 0-183 months). The median overall survival was 11.0 months (95% CI: 4.4–17.6) for patients with primary tumors and 7.0 months (95% CI: 5.0–9.0) for those with metastatic tumors. Although the log-rank test did not reveal a significant difference in survival between the two groups (*P* = 0.53), multivariable Cox regression analysis similarly showed no significant association between tumor origin and survival (HR: 0.838; 95% CI: 0.607–1.157; *P* = 0.28).

 Among the primary liver tumor cases, 28 (63.6%) of 44 HCC cases died (follow-up period: 1-80 months), while in 48 cholangiocarcinoma cases, 43 (89.6%) had died (follow-up period: 1-80 months). The mean overall survival was 34.9 months for HCC patients, and 11.6 months for cholangiocarcinoma patients. One case of hepatoblastoma was alive with a follow-up period of 37 months. According to the log-rank test, cholangiocarcinoma patients had significantly lower survival than HCC patients (*P* < 0.001), and this finding was confirmed in multivariable Cox regression (HR: 2.06; 95% CI: 1.17–3.62; *P* = 0.012).

 In HCC cases, the 5-year overall survival rate was found to be 38.6%. The analysis revealed that HCC cases with a single tumor had significantly better overall survival compared to those with multiple tumors (log-rank test, *P* = 0.033). Multivariable Cox regression also confirmed that having a single lesion was associated with improved survival (HR: 0.36; 95% CI: 0.21–0.63; *P* < 0.001). When grouped by tumor size, among 36 patients with a tumor size < 10 cm, 23 (63.9%) had died, while in 8 patients with a tumor size ≥ 10 cm, 5 (62.5%) had died. The average survival for patients with tumors < 10 cm was 35.7 months, and their 5-year overall survival rate was 38.9%, while for those with tumors ≥ 10 cm, the average survival was 30.1 months, and the 5-year survival rate was 37.5%. The 1-year and 3-year survival rates for patients with tumors < 10 cm were 77.8% and 47.2%, respectively, while for those with tumors ≥ 10 cm, the 1-year and 3-year survival rates were 50% and 37.5%, respectively. However, no statistically significant difference was found in the overall survival rates between the two groups (log-rank test, *P* = 0.55). In Cox regression, increasing tumor diameter was significantly associated with poorer survival both as a continuous variable (HR: 1.46; 95% CI: 1.19–1.80; *P* < 0.001) and as a dichotomized variable ( ≥ 10 cm vs < 10 cm: HR: 21.67; 95% CI: 1.92–244.29; *P* = 0.013).

 In cholangiocarcinoma cases, those with a single tumor had significantly better overall survival compared to those with multiple tumors (log-rank test, *P* < 0.001). This result was supported by multivariable Cox regression, where multiple liver lesions were significantly associated with worse overall survival (HR: 0.30; 95% CI: 0.13–0.67; *P* = 0.003). The overall survival for cases with tumors < 10 cm and ≥ 10 cm were 15.5 months and 7.6 months, respectively. However, no statistically significant difference in overall survival was found between the two groups (log-rank test, *P* = 0.10). In Cox analysis, tumor size was also not significantly associated with survival (*P* = 0.87).

 For metastatic tumors, the average overall survival for cases with a single tumor was 44.3 months, while for those with multiple tumors, it was 26.4 months. The log-rank test showed a statistically significant difference between these groups (*P* = 0.033), but Cox regression did not confirm this finding (HR: 0.75; 95% CI: 0.49–1.15; *P* = 0.19). When grouped by tumor size using a 10 cm cutoff, the overall survival for patients with tumors < 10 cm was 32.9 months, while for those with tumors ≥ 10 cm, it was 15.9 months. A statistically significant difference in overall survival was also found between these two groups (log-rank test, *P* = 0.006). However, in Cox regression, dichotomized tumor size was not significantly associated with survival (HR: 0.85; 95% CI: 0.43–1.66; *P* = 0.63). When metastatic adenocarcinoma cases were grouped according to their origin, the average overall survival rates were as follows: 27.8 months for GI tract, 10.5 months for respiratory system, 4 months for pancreatobiliary system (PBS), and 89.1 months for breast origin, with overall survival rates of 15%, 0%, 2.2%, and 35%, respectively.

 In multivariable Cox regression analysis including age, sex, number of liver lesions, tumor size, and primary versus metastatic tumor status, several factors were significantly associated with overall survival. Increasing age (HR: 1.034; 95% CI: 1.022–1.046; *P* < 0.001) and male sex (HR: 1.619; 95% CI: 1.231–2.130; *P* = 0.001) were independently associated with shorter survival. Patients with multiple liver lesions had significantly worse survival compared to those with solitary lesions (HR: 0.575; 95% CI: 0.411–0.804; p = 0.001). Tumor size as a continuous variable showed a borderline association with survival (HR: 1.049; 95% CI: 1.000–1.100; *P* = 0.050). However, primary versus metastatic tumor origin (*P* = 0.28) and categorization by tumor diameter ( ≥ 10 cm vs < 10 cm; *P* = 0.90) were not significantly associated with survival.

###  Immunohistochemical Data

####  HCC

 In 38 of the 44 HCC cases, glypican-3 IHC staining was performed, and focal/diffuse positivity was observed in 29 cases (76.3%), while no staining was seen in 9 cases (23.7%). Arginase 1, HepPar, p-CEA (canalicular staining), and CD10 (canalicular staining) were evaluated as markers indicating hepatocyte differentiation. Arginase 1 was applied to 11 cases, and staining was absent in 3 cases (27.3%). HepPar was applied to 23 cases, with no staining in 3 cases (13%). Staining was found in all 28 HCC cases where p-CEA was applied. CD10 was applied to five cases, and no staining was observed in three cases (60%). However, a combination of these markers was used in most of the cases. In the cases where one of the markers indicating hepatocyte differentiation was negative, all other markers, except for two exceptional cases, were positive. In these cases, reduction of the normal reticulin framework was detected. In one of the exceptional cases, both arginase 1 and HepPar were negative, while glypican-3 and HSP70 were positive. In the other exceptional case, HepPar, CD10, and glypican-3 were negative. In these cases, the diagnosis of HCC was made by evaluating histopathological, clinical, radiological, and biochemical markers together. No markers for hepatocyte differentiation were applied in six cases but all of these cases showed reduction of the normal reticulin framework. In three of these cases, the tumor tissue was too small to test these markers. In the other three cases where no hepatocyte differentiation markers were applied, the tumor was well-differentiated and developed in a cirrhotic background, so hepatocytic markers were not required.

###  Cholangiocarcinoma, Pancreatic Adenocarcinoma, Colon Adenocarcinoma, Gastric Adenocarcinoma, and Lung Adenocarcinoma

 The IHC profiles of primary and metastatic tumors with adenocarcinoma morphology are summarized in Tables 3 and 4. In metastatic tumors, the IHC results were consistent with the clinically detected mass, and decision regarding the primary tumor was made by evaluating all these findings together. In cases exhibiting adenocarcinoma morphology with IHC results consistent with cholangiocarcinoma, those in which no other mass was detected on whole-body imaging were classified as primary cholangiocarcinoma.

**Table 3:** CK7 and CK20 Profiles of the Tumors

**Tumor Type**	**CK7**	**CK20**	**CK19**	**CK7**^+^**CK20**^+^	**CK7**^+^**CK20**^-^	**CK7**^-^**CK20**^+^	**CK7**^-^**CK20**^-^
Cholangiocarcinoma	43/47 (91.5%)	2/47 (4.3%)	39/39 (100%)	2/47 (4.3%)	41/47 (87.2%)	0/47 (0%)	4/47 (8.5%)
Pancreatic adenocarcinoma	37/40 (92.5%)	7/40 (17.5%)	26/26 (100%)	6/40 (15%)	31/40 (77.5%)	1/40 (2.5%)	2/40 (5%)
Colon adenocarcinoma	4/33 (12.1%)	30/33 (90.9%)	2/2	4/33 (12.1%)	0/33 (0%)	26/33 (78.8%)	3/33 (9.1%)
Stomach adenocarcinoma	4/6	1/6	1/1	1/6	3/6	0/6	2/6
Lung adenocarcinoma	15/16	2/14	6/6	1/14	12/14	1/14	0/14
Breast carcinoma	14/22	0/14	-	0/14	9/14	0/14	5/14
Female genital system	6/9	0/9	-	0/9	6/9	0/9	3/9

**Table 4:** CDX2, TTF1, and GATA-3 Profiles of the Tumors and Chromogranin A, Synaptophysin, and CD56 Staining Status of Neuroendocrine Neoplasms According to Their Origin

**Tumor Type**	**CDX2**		**TTF1**		**GATA-3**
Cholangiocarcinoma	13/44 (29.5%)		0/36		2/5 (focal)
Pancreatic adenocarcinoma	19/38 (50%)		0/15		1/4 (focal)
Colon adenocarcinoma	32/33 (97%)		0/12		0/2
Stomach adenocarcinoma	7/7		0/1		-
Lung adenocarcinoma	3/13		12/17 (70.6%)		0/1
Breast carcinoma	0/9		0/3		25/26 (96.2%)
Female genital	1/6		0/2		-
**Tumor Origin of NENs**	**Chromogranin A**	**Synaptophysin**	**CD56**	**CDX2**	**TTF1**
NEN of Respiratory system	11/19 (57.9%)	19/22 (86.4%)	20/21 (95.2%)	0/2	16/25 (64.0%)
NEN of GI and PB system	5/6	6/6	2/3	2/3	1/3

NEN: Neuroendocrine neoplasm, GI: Gastrointestinal, PB: Pancreatobiliary.

###  Breast Carcinoma

 Among 40 adenocarcinoma cases originating from the breast, the breast biopsy records were available for 31 cases in the hospital archives before the liver biopsy. The CK7 and CK20 profiles of the tumors in the liver core biopsies are summarized in Table 3. Additionally, all cases were tested with important therapeutic markers such as ER, PR, HER2, and at least one of the breast-specific markers (GATA-3, TRSP-1, GCDFP-15, Mammaglobin). The breast-specific markers were as follows: GATA-3 was positive in 25 out of 26 cases (96.2%) with diffuse strong positivity; TRSP-1 was positive in all three cases (100%); GCDFP-15 was positive in 10 out of 28 cases (35.7%); and Mammaglobin was positive in 12 out of 28 cases (42.9%). ER ranged from 0-95%, with an average of 60.2% and a median of 70%. ER was > 1% positive in 33 out of 40 cases (82.5%). PR ranged from 0-90%, with an average of 11.1% and a median of 0%. PR was > 1% positive in 11 out of 40 cases (27.5%). HER2 was positive ( + 3) in 9 out of 40 cases (22.5%), suspicious ( + 2) in 8 cases (20%), and negative (0 and + 1) in 23 cases (57.5%).

 When comparing data from the previous breast biopsy with those from the liver biopsy in 31 cases, it was observed that in two cases (6.5%), the ER status changed from positive ( > 1%) to negative ( < 1%). PR changed from positive ( > 1%) to negative ( < 1%) in 10 cases (32.3%), and in one case (3.2%), it changed from negative ( < 1%) to positive ( > 1%). HER2 was consistent with the previous breast biopsy in 29 out of 31 cases (93.5%). In one case, it changed from positive to negative, and in another, it changed from negative to positive.

###  Female Genital System

 Of the 10 tumors originating from the female genital system, 6 were serous carcinoma, 3 were endometrioid adenocarcinoma, and one was squamous cell carcinoma. The CK7 and CK20 profiles of the tumors are summarized in Table 3.

###  Neuroendocrine Neoplasms 

 Of the 32 poorly differentiated neuroendocrine carcinomas (NECs) cases, 25 originated from the lungs, one from the pancreas, one from the colon, one from the skin, and the primary site of 4 tumors could not be determined. Among the six well-differentiated neuroendocrine tumors (NETs) cases, three were pancreatic, one was colonic, one was gastric, and the primary site of one case was unknown. When evaluating the IHC profile of the total 38 cases diagnosed with NEC and NET, the following results were observed: CK AE1/AE3 was positive in 21 of 24 cases (87.5%), CK7 was positive in 10 of 19 cases (52.6%), CK20 was positive in one of 13 cases (7.7%), chromogranin A was positive in 21 of 30 cases (70%), synaptophysin was positive in 30 of 33 cases (90.9%), CD56 was positive in 26 of 28 cases (92.9%), CDX2 was positive in 4 of 7 cases (57.1%), and TTF1 was positive in 19 of 32 cases (59.4%). The CDX2, TTF1, chromogranin, synaptophysin and CD56 staining status of NENs according to their localization of origin are shown in Table 4.

###  TTF1, CDX2, and GATA-3

 Excluding tumors of pulmonary origin, TTF1 was found to be positive in only 4 out of 72 cases (5.6%). Of these four cases, three were NENs, and one was a carcinoma of unknown primary. For adenocarcinomas, excluding NENs, TTF1 positivity was highly useful in identifying pulmonary origin. When tumors of unknown primary were excluded, only one out of 43 cases (2.3%) was positive, which was a colon-origin NET case.

 Excluding tumors of unknown primary, GI, and PBS origin, CDX2 was positive in only 4 out of 38 cases (10.5%). These four cases included three lung adenocarcinomas and one ovarian-origin endometrioid adenocarcinoma.

 Excluding tumors of mammary and urinary system origin, GATA-3 was applied to 11 tumors, and focal positivity was observed in only one case. None of the tumors showed diffuse strong staining.

## Discussion

 In our study, we observed that malignancy is a common diagnosis in patients with radiologically defined liver masses, regardless of a known history of oncological malignancy, and liver metastases were more common than primary tumors. One of the notable findings of this study was identification of malignancy for the first time in 64 patients who underwent liver biopsy from a mass lesion. Excluding the seven lymphoma cases, in 189 (74.7%) out of 253 metastatic cases, the primary tumor was already known either pathologically or radiologically at the time of liver biopsy. Interestingly, we identified 64 cases (25.3%) with liver masses, in which the tumor was thought to be metastatic, requiring further investigation to determine the primary source, despite no previous history of malignancy. After liver biopsy, the primary tumor was found in 23 (36%) of these 64 cases. The primary tumors identified in these 23 cases were: 9 lung, 7 colon, 3 stomach, 2 pancreas, one ovary, and one gallbladder metastasis. Notably, in 10 of 48 tumors (20.8%) originating from the the GI tract and in 9 of 50 (18%) tumors related to the respiratory system, the primary tumor could be determined radiologically only after liver biopsy. This may be due to abdominal imaging methods being relatively less sensitive for detecting GI tract tumors. The high rate of tumors originating from the lung may be attributed to the fact that thoracic examination was not performed in addition to abdominal imaging when liver masses were detected. However, tumors originating from abdominal organs other than the GI tract were largely detected during biopsy via abdominal imaging methods. These results suggest that cases with liver masses that have IHC results consistent with GI tract tumors on biopsy but no primary tumor on abdominal imaging should be further investigated with endoscopic procedures.

 An important finding of our study is the recommendation to continue follow-up and perform a new biopsy in cases where no neoplastic lesion is detected in the initial biopsy of radiologically identified liver masses. In cases where the initial biopsy reveals normal liver tissue, inflammation, or cirrhosis, a second biopsy was performed in eight patients, and malignancy was diagnosed in six (75%) of these cases with the second biopsy.

 In our study, lung, pancreas, breast, and colon-originated tumors were found to be the most common metastatic tumors. This finding is consistent with the literature.^2,3^ While much of the current literature has focused on specific primary tumors and their tendency to metastasize to the liver, there is limited data regarding liver metastasis in patients with different primary malignancies. Our study highlights the distribution of liver metastases observed in a cohort of patients with radiologically newly detected liver masses, without selecting a specific primary origin.

 It has been reported that metastatic tumors in the liver are frequently seen as multifocal nodular lesions; however, they can also present as a single mass or conglomerate masses.^5^ In our study, multiple masses were observed in 53.3% of primary tumors, while 12% of metastatic tumors presented as a single mass. As expected, the likelihood of multiple metastatic tumors was found to be statistically significantly higher. However, considering that more than half of primary tumors were multifocal, one should be cautious about immediately assuming metastasis in the presence of multiple masses and should keep in mind that the tumor could be primary. Similarly, it should be also remembered that metastatic tumors can present as a single mass. This finding was also supported by Cox regression analysis, where tumor multiplicity was significantly associated with poorer survival in primary liver tumors, and specifically in cholangiocarcinoma cases.

 In HCC cases, the 5-year overall survival rate was found to be 38.6%. Large HCC tumors ( > 10 cm) are frequently associated with poorer prognosis in the literature.^6^ In our study, it was found that HCC cases with a single mass had statistically significantly longer overall survival compared to those with multiple masses. Multivariable Cox regression also revealed that increasing tumor diameter and tumor size ≥ 10 cm were independent predictors of poorer survival in HCC patients. However, although solitary tumors had a trend toward better survival, this did not reach statistical significance.

 The prognosis of unresectable intrahepatic cholangiocarcinoma is generally poor, with a median survival time of 6-9 months. Complete resection remains the only hope for long-term recovery; however, the 5-year overall survival rate after hepatectomy is approximately 30%.^7^ In our study, the mean overall survival of cholangiocarcinoma cases was 11.6 months, and the 5-year overall survival rate was 10.4%. In Cox regression analysis, tumor multiplicity was the only factor significantly associated with poor survival in cholangiocarcinoma patients. Other variables such as sex, age, and tumor size were not significant predictors.

 It is well known that patients with metastatic lesions in the liver have poor survival rates. In our study, the mean overall survival for patients with metastatic tumors was 23.4 months, and the overall survival rate was only 12.3%. It was observed that patients with breast cancer metastasis had significantly better mean overall survival compared to adenocarcinomas originating from the GI tract, respiratory system, and PBS. The literature reports the average overall survival of metastatic breast cancer patients at 2-3 years.^8^ However, our study found a much longer mean overall survival of 83.1 months. It is thought that factors such as hormone receptor status and evolving treatment algorithms may have contributed to the increased survival times in metastatic breast cancer. Additionally, Cox regression analysis showed that male sex and older age were independently associated with decreased survival in metastatic tumor cases, whereas tumor size and multiplicity were not statistically significant.

 As stated in the review by Vyas and Jain, the staining rates for HepPar-1, arginase-1, pCEA (canalicular), CD10 (canalicular), and glypican-3 in HCCs are reported to be 70-84%, 44-89%, 45-81%, 50-74%, and 89%, respectively.^9^ In our study, the positivity rates for these markers were found to be 87%, 72.7%, 100%, 40%, and 76.3%, respectively. However, despite having 44 HCC cases, the small number of cases stained with arginase-1 and CD10 could be considered a limitation of our study. Our data indicate that pCEA is the most sensitive marker for showing hepatocytic differentiation in HCCs. Both the literature publications and our data suggest that there may be HCCs that do not stain with these markers. Therefore, in cases where hepatocytic differentiation is questionable, it would be more appropriate to use a combination of multiple markers rather than relying on a single marker. Additionally, due to the low sensitivity of glypican-3 in detecting malignancy, especially in well-differentiated HCCs^10^, HCC should not be definitively excluded based solely on glypican-3 negativity.

 In tumors exhibiting adenocarcinoma morphology, markers such as CK7, CK19, CK20, CDX2, TTF1, GATA-3, PAX8, TRSP-1, GCDFP-15, Mammaglobin, NKX3.1, and PSAP are utilized for distinguishing primary cholangiocarcinoma from metastatic adenocarcinomas and for identifying the primary origin of metastatic tumors. Traditionally, cholangiocarcinomas, lung adenocarcinomas, gastric adenocarcinomas, pancreatic adenocarcinomas, and breast carcinomas are known to be CK7 ^+^ CK20 -, while colon adenocarcinomas are CK7^-^ CK20^+^, and a portion of gastric and pancreatic adenocarcinomas are CK7^+^ CK20^+^. However, the literature also reports cases with abnormal CK7 and CK20 immunophenotypes, though these are less frequent.^11^

 Cholangiocarcinomas are typically positive for CK7 and CK19. Additionally, a subset of cases may also express CK20 and CDX2. In our study, 91.5% of cholangiocarcinoma cases were positive for CK7, and 100% were positive for CK19. CK20, which is important for differentiating from metastatic adenocarcinomas, was positive in 4.3% of cases. The CDX2 positivity found in 29.5% of our cholangiocarcinoma cases is similar to the 29% CDX2 positivity reported by Tang et al.^12^

 In our study, 77.5% of pancreatic adenocarcinomas, 50% of gastric adenocarcinomas, 85.7% of lung adenocarcinomas, and 64.3% of breast carcinomas exhibited a CK7^+^ CK20^-^ immunophenotype. Also, 16.7% of gastric adenocarcinomas and 15% of pancreatic adenocarcinomas showed a CK7^+^ CK20^+^ immunoprofile, and 78.8% of colon adenocarcinomas were found to be CK7^-^ CK20^+^. These findings are consistent with the literature, and, similar to data from other studies, cases exhibiting an abnormal CK7 and CK20 profile were also identified in our study. A small subset of poorly differentiated CRCs may show loss of CDX2 and/or CK20 expression. Therefore, it should be remembered that CDX2 - CK20 - CRCs can metastasize to the liver.^13^ Notably, a significant proportion of breast carcinoma (35.7%) and gastric adenocarcinoma (33.3%) cases were CK7^-^ CK20^+^, which suggests that this finding could be due to changes in the immunoprofile of metastatic tumors or expression loss related to treatment effects.

 It has been reported in the literature that the status of ER, PR, and HER2 can vary in metastatic breast carcinoma.^14^ In a comprehensive review study, the median discordance rates between primary tumors and metastases for ER, PR, and HER2 expression were reported at 14%, 21%, and 10%, respectively.^15^ In the study by Broom et al, the discordance rates for these markers were 17.7%, 37.3%, and 5.5%, respectively.^14^ In our study, the discordance rates for ER, PR, and HER2 were found to be 6.5%, 35.5%, and 3.2%, respectively.

 In our study, the most useful marker for identifying breast carcinoma was GATA-3. GATA-3, which was positive in 96.2% of the cases, showing higher success compared to GCDFP-15 and mammaglobin. Similarly, in the literature, GATA-3 has been reported to be superior to GCDFP-15 and mammaglobin in identifying breast carcinoma in metastatic tumors.^16,17^ However, GATA-3 has low specificity due to significant positivity in tumors like bladder carcinoma, and this should be kept in mind when attempting to identify the primary tumor in metastatic cases.^17^

 In small cell lung carcinomas, the positivity rates for chromogranin A, synaptophysin, and CD56 are reported at 47%, 67%, and 97%, respectively.^18^ In our study, these rates were found to be 57.9%, 86.4%, and 95.2% for NENs originating from the respiratory system. Particularly noteworthy was the fact that only 11 out of 19 respiratory system-originating tumors showed positivity for chromogranin A. However, in our study, synaptophysin appeared to be a better marker, with a positivity rate of 86.4%. These data suggest that chromogranin A should be used as the third choice after synaptophysin and CD56 in NENs originating from the respiratory system. CD56 is primarily used as a neuroendocrine marker in the diagnosis of lung NENs, but it should be interpreted with caution and never used as a single marker.^19^ In our study, although high positivity was found in respiratory system-originating tumors, the positivity rate was 66.7% for GI and PBS-originating tumors, which was lower compared to chromogranin A and synaptophysin. While the data in our study is consistent with literature suggesting that chromogranin A and synaptophysin should be preferred for GI and PBS tumors, the fact that only three cases were tested for CD56 in our study prevents us from making a definitive conclusion regarding this marker.

 TTF1 is generally considered to be highly specific for broncho-pulmonary NETs, although there are many reports in the literature regarding TTF1 positivity in extrapulmonary NETs. In contrast, TTF1’s sensitivity for lung-origin lesions is not as impressive, and it shows considerable variability in the existing literature; for pulmonary carcinoid tumors, the sensitivity ranges from 43% to 69%, while for small cell carcinomas, it can reach up to 90%.^20^ However, it is not specific for pulmonary origin in metastatic small cell carcinoma.^21,22^ Additionally, a positivity rate of approximately 9.5% for CDX2 has been reported in metastatic small cell lung carcinomas.^13^ Therefore, determining the primary origin in metastatic NENs using organ-specific transcription factors like TTF1 and CDX2 is almost always impossible and requires clinical correlation.^19^ In our study, TTF1 was positive in only 64% of 25 respiratory system-originating tumors, while 3 out of 7 tumors originating from other systems were positive. These results suggest that TTF1 and CDX2 are not highly reliable markers for identifying the primary tumor in NENs.

 When excluding tumors of respiratory system origin, TTF1 was found to be positive in only 4 out of 72 cases (5.6%). TTF1 is considered a highly useful marker for identifying lung origin in adenocarcinomas. However, it is noteworthy that 29.4% of the 17 lung adenocarcinomas in our study were negative for TTF1. Excluding tumors of unknown primary origin, GI and PBS-originating tumors, and cholangiocarcinomas, CDX2 was positive in only 4 out of 38 cases (10.5%). These findings not only demonstrate the utility of TTF1 and CDX2 in determining the primary origin, particularly in adenocarcinomas, but also highlight the possibility of tumors with rare abnormal expression. Therefore, histopathological and IHC findings should be correlated with clinical and radiological data when determining the primary tumor.

## Conclusion

 Liver masses pose diagnostic challenges, requiring differentiation between primary and metastatic lesions. This study underscores the critical role of liver biopsy, alongside radiological and clinical findings, in identifying malignancies and determining metastatic origins. While IHC markers aid in distinguishing primary tumors from metastases, their use should be integrated with other diagnostic tools for accuracy. Further studies are needed to refine these techniques and improve diagnostic precision.
